# Modeling the Prader–Willi Syndrome-Associated Growth Phases in Snord116 KO Mice

**DOI:** 10.3390/ijms27209344

**Published:** 2026-10-21

**Authors:** Delf-Magnus Kummerfeld, Tasneem Khanam, Anna Wolters, Boris V. Skryabin, Timofey S. Rozhdestvensky

**Affiliations:** 1 Medical Faculty, Core Facility Transgenic Animal and Genetic Engineering Models (TRAM), University of Muenster, Von-Esmarch-Str. 56, 48149 Muenster, Germany; 2 Leibniz Institute on Aging—Fritz Lipmann Institute (FLI), 07745 Jena, Germany; 3 Department of Neurology, University Hospital Essen, University of Duisburg-Essen, Hufelandstr. 55, 45122 Essen, Germany

**Keywords:** Prader–Willi syndrome (PWS), SNORD116, postnatal growth, body-weight regulation, snoRNA, mouse model, animal model of human disease

## Abstract

Prader–Willi syndrome (PWS) is a neurodevelopmental disorder characterized by distinctive developmental transition from neonatal hypotonia and poor feeding to progressive weight gain and, later, hyperphagia and obesity. The paternally expressed SNORD116 cluster is critical for postnatal growth, but how its deficiency impacts the progression of PWS remains unclear. Here, we analyzed the day-over-day growth rates for 351 mice with Snord116 deletion or altered Snord116 dosage, including their wild-type littermates, across both sexes and two genetic backgrounds. Snord116 expression increased markedly during early brain development. The Snord116-deficient mice exhibited a distinct three-stage growth trajectory: impaired growth until approximately P16, normalization between P17 and P21, followed by transient accelerated weight gain until approximately P30. Although growth rates subsequently normalized, the accumulated deficit was not fully compensated, and the adult Snord116KO mice remained smaller than the wild-type controls. These developmental transitions broadly parallel the early nutritional phases of human PWS, although the mice did not progress to exhibit persistent obesity-associated growth. Reactivation of the maternal Snord116 cluster partially rescued the growth phenotype, whereas activation of both parental alleles produced a modest growth advantage, which was most evident in the males. These findings identify early postnatal development as a critical window of Snord116-dependent growth regulation and provide a framework for investigating the mechanisms underlying PWS’s progression.

## 1. Introduction

Prader–Willi syndrome (PWS; OMIM #176270) is a rare, neurodevelopmental disorder caused by the loss of expression of paternally inherited, imprinted genes within the chromosome 15q11–q13 locus. The clinical phenotype encompasses hypotonia, developmental delay, characteristic behavioral abnormalities, endocrine dysfunction, altered body composition, impaired growth, and profound disturbances of appetite and energy homeostasis [1]. A defining feature of PWS is the marked developmental progression of its nutritional and weight phenotype. Individuals with PWS typically undergo a progression of distinct changes in feeding behavior and body-weight regulation, beginning with severe feeding difficulties and poor growth in infancy and progressing toward excessive weight gain, hyperphagia, and obesity later in childhood [1,2,3,4,5].

Multiple clinical studies have established a series of nutritional phases that describe this developmental progression [2,3,4]. During the early postnatal period, individuals with PWS commonly exhibit hypotonia, weak sucking ability and, as a result, poor feeding, and reduced weight gain. As feeding abilities improve, growth becomes more stable, followed by a transition toward increasing body weight. Importantly, the initial phase of excessive weight gain is not necessarily associated with a concurrent increase in appetite or food intake, preceding the later onset of hyperphagia and impaired satiety [2,4]. Changes in the regulation of body-weight gain represent an early and dynamic component of the PWS phenotype and are not merely a consequence of increased food consumption [6]. The progressive nature of these changes suggests that the mechanisms controlling growth, energy expenditure, body composition, appetite, and satiety are likely to be differentially affected during development [6,7,8]. This developmental progression in PWS is, therefore, relevant for understanding its underlying molecular mechanisms. A conventional representation of body-weight change over the time course of an experiment provides only a cumulative representation of growth, but does not represent changes in the underlying growth rate. By contrast, analysis of the relative change in body weight between consecutive day-over-day measurements provides a better understanding of the dynamics of growth and may reveal transient developmental stages that are not readily apparent from the absolute body weight alone. Identifying such transitions is important because molecular processes contributing to the PWS phenotype may be developmentally regulated both during embryogenesis as well as postnatally, through the early stages of life [6,9].

**Figure 1:**
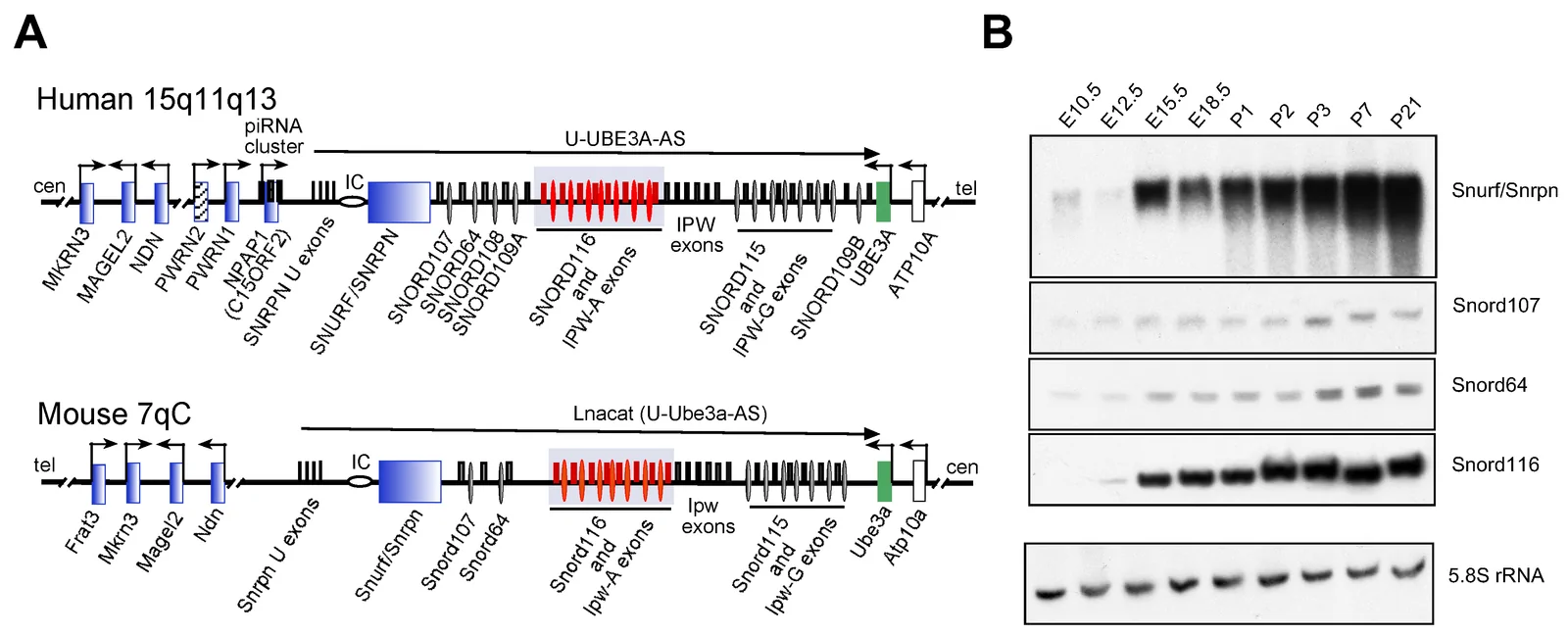
Organization of the human and mouse PWS loci and developmental expression of the corresponding mouse transcripts. (**A**) Schematic representation of the human and mouse PWS regions on chromosomes 15q11–q13 and 7qC, respectively. The imprinting center (IC) is represented by a horizontal oval and blue rectangles indicate paternally expressed protein-coding genes. Thin ovals indicate snoRNA genes, while the thin rectangles above the midline denote non-protein-coding exons. SNORD116 and IPW-A exons are highlighted in red and enclosed by a grey rectangle. Arrows indicate promoters and the direction of transcription. The long arrow at the top represents the putative U-UBE3A antisense transcript (U-UBE3A-AS), which encompasses the snoRNA genes as well as the SNORD116 and SNORD115 clusters. The centromeric and telomeric directions are indicated as cen and tel, respectively. (adapted from [40]). (**B**) Northern blot analysis of Snord116 and upstream transcripts during mouse embryonic and early postnatal development. Expression of Snurf/Snrpn, Snord64, Snord107, and Snord116 was analyzed, as described in the Materials and Methods section. Membranes detecting Snurf/Snrpn, Snord64, and Snord116 were exposed at −80 °C for 4 days; the Snord107 membrane was exposed for 10 days, and the 5.8S rRNA membrane for 4 h. 5.8S rRNA was used as a loading control.

The organization and imprinting of the PWS-associated regions are conserved between human chromosome 15q11–q13 and the corresponding region of mouse chromosome 7 (Figure 1A). The paternal domain contains several paternally expressed protein coding genes and non-coding RNAs, including the SNORD116 and SNORD115 small nucleolar RNA (snoRNA) clusters [10,11,12,13]. These snoRNA clusters are embedded within introns of the large, paternally expressed UBE3A-ATS (Ube3a-antisense transcript) and are transcribed as part of this extended non-coding transcriptional unit [11,13]. In neurons, UBE3A-ATS contributes to the regulation of the imprinted UBE3A locus [14,15,16,17], while the specific molecular functions of SNORD116 and SNORD115 remain elusive [18,19,20,21,22,23,24,25]. Early genetic studies in mice and patients with chromosomal rearrangements implicated the SNORD116 gene cluster as the PWS critical region (PWScr) [26,27,28,29]. In 2007, we selectively deleted the Snord116 cluster in mice, demonstrating that the paternal, but not maternal, deletion (PWScr^p−/m+^) causes pronounced postnatal growth retardation [30]. This phenotype was observed across different genetic backgrounds, notably, around 15% of postnatal lethality was also detected, but only in the C57BL/6J background. An independent model with a slightly larger deletion reproduced the growth phenotype and suggested additional PWS-related abnormalities [31]. The relevance of the SNORD116 array region to human PWS was subsequently supported by the discovery of patients carrying paternal microdeletions encompassing the SNORD116 cluster, beginning with the report by Sahoo et al. (2008) [32] and followed by additional cases with overlapping deletions of different sizes [33,34,35,36,37]. In the subsequent study we demonstrated that transcriptional activation of the normally silent maternal expression of non-coding RNAs (ncRNAs) from the Snord116 locus could partially rescue early postnatal growth retardation, with body weight approaching wild-type levels by, approximately, postnatal day 21 [38]. Together, these genetic studies established the Snord116 cluster as an important regulator of postnatal growth. Notably, attempts to rescue the growth phenotype by generating transgenic mouse models expressing Snord116 alone or in combination with portions of its host Ube3a-AS transcript have so far failed to reproduce this rescue phenotype [31,38,39] (Kummerfeld et al., in preparation), suggesting that, in addition to appropriate expression levels, proper transcript processing, as well as the spatial context of expression within certain brain compartments, may be critical for the growth-regulatory function of Snord116-cluster-derived ncRNAs [31,38,39].

In the present study, we performed an extended analysis of previously generated longitudinal body-weight data from 351 mice, including Snord116 knockout (PWScr^p−/m+^, in the following context designated as Snord116KO) mice and their wild-type littermates (Table 1) at daily resolution [30,38]. Rather than focusing exclusively on absolute body weight, we focused on the day-over-day growth rate (i.e., relative change in body weight between consecutive days) to characterize the developmental dynamics. We further examined these trajectories in both sexes and across two genetic backgrounds to assess the robustness of the observed developmental pattern. Our data revealed that distinct growth phases are detectable in Snord116 KO mice, including an initial period of impaired growth, subsequent normalization, and a phase of accelerated growth, followed by normalization of growth rate in adulthood. In addition, we applied the same analysis of body weight data to PWScr^p−/m+5′LoxP^ mice, in which the normally silent maternal Snord116 cluster is transcriptionally activated, providing a genetic model of putative maternal compensation of the PWS growth phenotype [38]. Importantly, we also present, for the first time, longitudinal growth data from a mouse model in which both the paternal and maternal Snord116 alleles are transcriptionally active (PWScr^p+/m+5′LoxP^), enabling assessment of the developmental progression changes resulting from an increased dosage of Snord116-cluster ncRNAs. Together, these analyses provide a comprehensive description of the temporal progression of the postnatal Snord116-associated growth phenotype and allow us to distinguish developmental patterns associated with the loss, maternal reactivation, and increased dosage of Snord116-cluster expression. This approach provides a framework for correlating the phenotype of the mouse model to the respective established developmental progression of human PWS and for defining critical developmental periods for subsequent molecular analyses.

## 2. Results

### 2.1. Developmental Expression of Non-Protein-Coding RNAs from the PWS Locus

In our previous studies, we characterized the developmental expression of Snord115 and Ipw RNAs, and demonstrated that these non-coding RNAs are developmentally regulated during embryonic and postnatal brain development [30]. Based on these observations, we extended the analysis to include the Snord116 cluster and upstream transcripts within the Snurf/Snrpn-Ube3a-ATS transcriptional unit (Figure 1A). We examined the expression of Snord107, Snord64 and Snord116 transcripts, together with the Snurf/Snrpn portion of the Ube3a-ATS transcript, across embryonic and postnatal developmental stages (Figure 1B).

This analysis was designed to determine the transcriptional-regulation window and characterize the temporal expression of the Snord116 array and its upstream transcripts. Expression was analyzed at E10.5, E12.5, E15.5 and E18.5 during embryonic development and at P1, P2, P3, P7, and P21 during the postnatal period. Notably, for E10.5 and E12.5, total RNA was isolated from whole embryos, whereas at E15.5, E18.5 and all postnatal stages, RNA was isolated from the entire brain. Previous RT-PCR analyses did not detect snoRNAs expression at E9.5, whereas expression became detectable at E10.5, in contrast to Snurf, which was already detectable at E9.5 [41]. We detected broadly similar developmental expression profiles for the Snurf/Snrpn transcript and Snord107, Snord64 and Snord116 snoRNAs. All four transcripts were barely detectable at E10.5 and E12.5 and showed a progressive increase during development, with a pronounced increase during the early postnatal period. Thus, the regulation of expression observed for the Snord116 region is consistent with the pattern previously observed for Snord115 and Ipw, extending the evidence for coordinated developmental regulation of the Ube3a-ATS host-transcript-derived non-coding RNAs within the mouse PWS locus. The parallel expression profiles are also consistent with the genomic organization of these transcripts. Snord107, Snord64, Snord116 and Snord115 are embedded within the larger Snurf/Snrpn-Ube3a-ATS transcriptional unit, and their coordinated increase may, therefore, reflect increasing transcriptional activity across this region and coordinated developmental regulation during mouse neurodevelopment. The observed timeline demonstrates that transcription is initiated during embryonic neurogenesis and strongly increases during the critical window of postnatal neuronal maturation [41,42].

### 2.2. Developmental Progression of Daily Relative-Body-Weight Changes in the Snord116KO PWS Mouse Model

**Figure 2:**
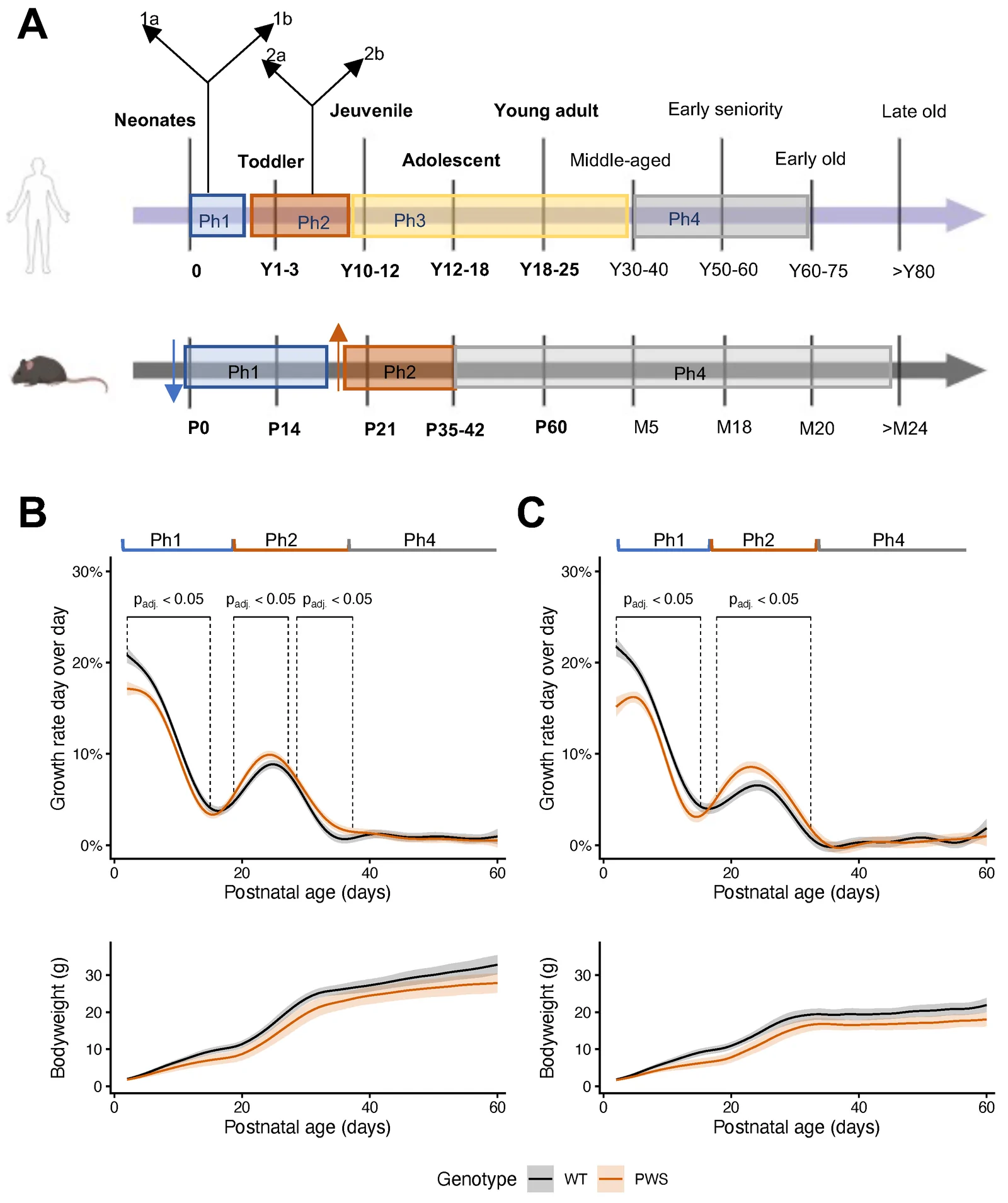
Correspondence between human and mouse developmental stages and growth dynamics in Snord116 knockout mice. (**A**) Schematic representation of the correspondence between human and mouse postnatal developmental stages, based on published lifespan and aging comparisons [43,44,45,46]. Clinical nutritional phases of Prader–Willi syndrome (PWS) are indicated by colored rectangles, with phases 1–4 denoted as Ph1, Ph2, Ph3, and Ph4, respectively [2,4]. The corresponding developmental intervals in mice (Ph1, Ph2 and Ph4), defined based on transitions in postnatal day-over-day growth rate, are indicated on the mouse-lifespan scale. (**B**,**C**) Growth dynamics in male (**B**) and female (**C**) Snord116KO (PWS) and wild-type (WT) mice on the FVB/N × C57BL/6J genetic background. Upper panels show the relative day-over-day change in body weight (growth rate), whereas lower panels show longitudinal body-weight trajectories. Shaded areas represent the corresponding 95% confidence intervals for growth rate, respectively, ±standard deviation (SD) of the mean for bodyweight. Intervals in which differences in growth rates between genotypes were statistically significant (*p*_adj_ < 0.05) are indicated by dashed lines.

To define the temporal development of the PWS-associated phenotype, we reanalyzed our previously generated growth data from Snord116 knockout mice (PWScr^p−/m+^) and their WT littermates from the early postnatal period through later development (Figure 2). These data originate from our previous studies of PWS mouse models, including the Snord116 knockout (Snord116KO) and the 5′LoxP model (PWScr^p-/m+5′LoxP^), in which we transcriptionally activated the Snord116 cluster from the otherwise silent maternal allele, resulting in a partial compensation for the growth phenotype observed in Snord116KO mice (Table 1). For the present analysis, at first, longitudinal growth data from Snord116KO mice and their WT littermates on two genetic backgrounds, C57BL/6J and FVB/N × C57BL/6J (50%/50%), were recalculated as the day-over-day growth rate (the percentage change in body weight relative to the preceding day of measurement) (Figure 2B,C). This analysis revealed distinct, postnatal age-dependent phases in the growth trajectory of the Snord116KO mice rather than a uniform growth retardation. During the first phase (approximately two postnatal weeks, P2–P16), the Snord116KO mice of both genders and genotypes exhibited a noticeably impaired growth rate compared to their WT littermates (Figure 2).

This early-growth impairment is consistent with the pronounced neonatal and early-infantile-growth phenotype characteristic of PWS in humans (Figure 2A). The next transition phase was evident in both sexes and for both genetic backgrounds ~ P17 and P21, during which the daily relative body-weight gain of the Snord116KO and WT mice became comparable (Figure 2B,C and Figure S1A,B).

**Figure 3:**
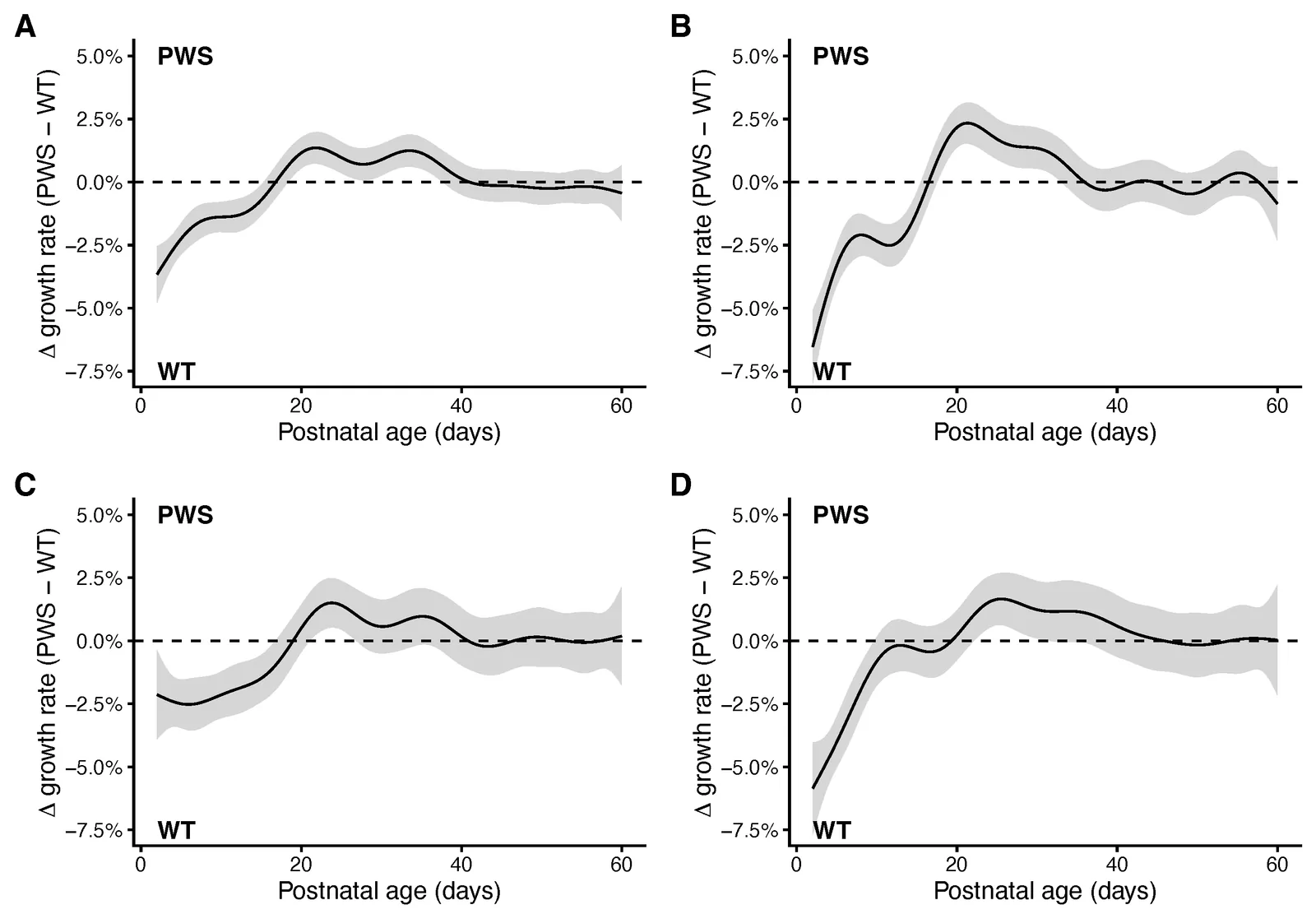
Differences in growth rate between Snord116KO (PWS) and wild-type mice. The difference in daily growth rate between Snord116KO (PWS) and wild-type (WT) mice is shown across postnatal development from P2 to P60. Male (**A**) and female (**B**) mice from the FVB/N × C57BL/6J genetic background. Male (**C**) and female (**D**) mice from the C57BL/6J genetic background. The solid black line represents the calculated difference between the fitted models of Snord116KO mice and WT mice growth rates (Δ growth rate; PWS − WT), with positive values indicating a higher growth rate in Snord116KO mice and negative values indicating a lower growth rate relative to WT mice. The grey shaded area represents the corresponding 95% confidence interval. The dashed horizontal line indicates no difference (Δ growth rate = 0) between genotypes. The temporal pattern of growth-rate differences was used to define developmental windows for comparison with the clinical phases of PWS.

This temporary convergence in growth velocity is particularly notable because it preceded the subsequent increase in the rate of body-weight gain observed in the Snord116KO mice. Around P21, the Snord116KO mice showed a marked change in growth, entering a period of sustained and significantly higher growth rates compared with the WT animals (Figure 3). This difference remained statistically significant until approximately P33 for the mice with the FVB × C57BL/6J background (Figure 2B,C and Figure S2) and until approximately P26 for the mice with the C57BL/6J background (Figures S1A,B and S2), thereby defining a distinct developmental window of accelerated body-weight gain in the Snord116KO mice. After ~ P40, the daily relative body-weight gain of the Snord116KO mice again stabilized, and was indistinguishable from that of the WT animals in either genotype (Figure 2B,C, Figure 3 and Figure S1A,B). Interestingly, the increased weight-gain phase observed between approximately P21 and P30 did not develop into a continuously progressive phenotype and instead reached a plateau. We further investigated for the possibility that the impaired growth of Snord116 KO mice is influenced by competition with their WT littermates. To address this question, we re-fitted the generalized additive mixed models (GAMMs), including litter as an additional random effect alongside the mouse-specific random intercept and compared this with the original models. The inclusion of litter did not improve the model fit (ANOVA comparisons, all *p* ≥ 0.9909), and the estimated litter-level variance was negligible across both genetic backgrounds and sexes. Thus, our analysis provides no evidence that within-litter competition or other litter-associated effects substantially contribute to the impaired growth of the Snord116 KO mice. Furthermore, mean litter size was smaller for the C57BL/6J mice (6.75 ± 3.08) than for the FVB/N × C57BL/6J mice (8.38 ± 2.66), yet the genotype-associated growth phenotype was comparable or more pronounced in the latter’s background. This observation further argues against litter size as a probable explanation for the observed phenotype. To our knowledge, this is the first direct statistical evaluation of the contribution of litter-level variation to the growth phenotype in Snord116 KO cohorts. Our findings exclude the possibility that the pronounced genotype-associated growth differences are due to litter effect.

### 2.3. Maternal Snord116 Activation Modulates Postnatal Growth

**Figure 4:**
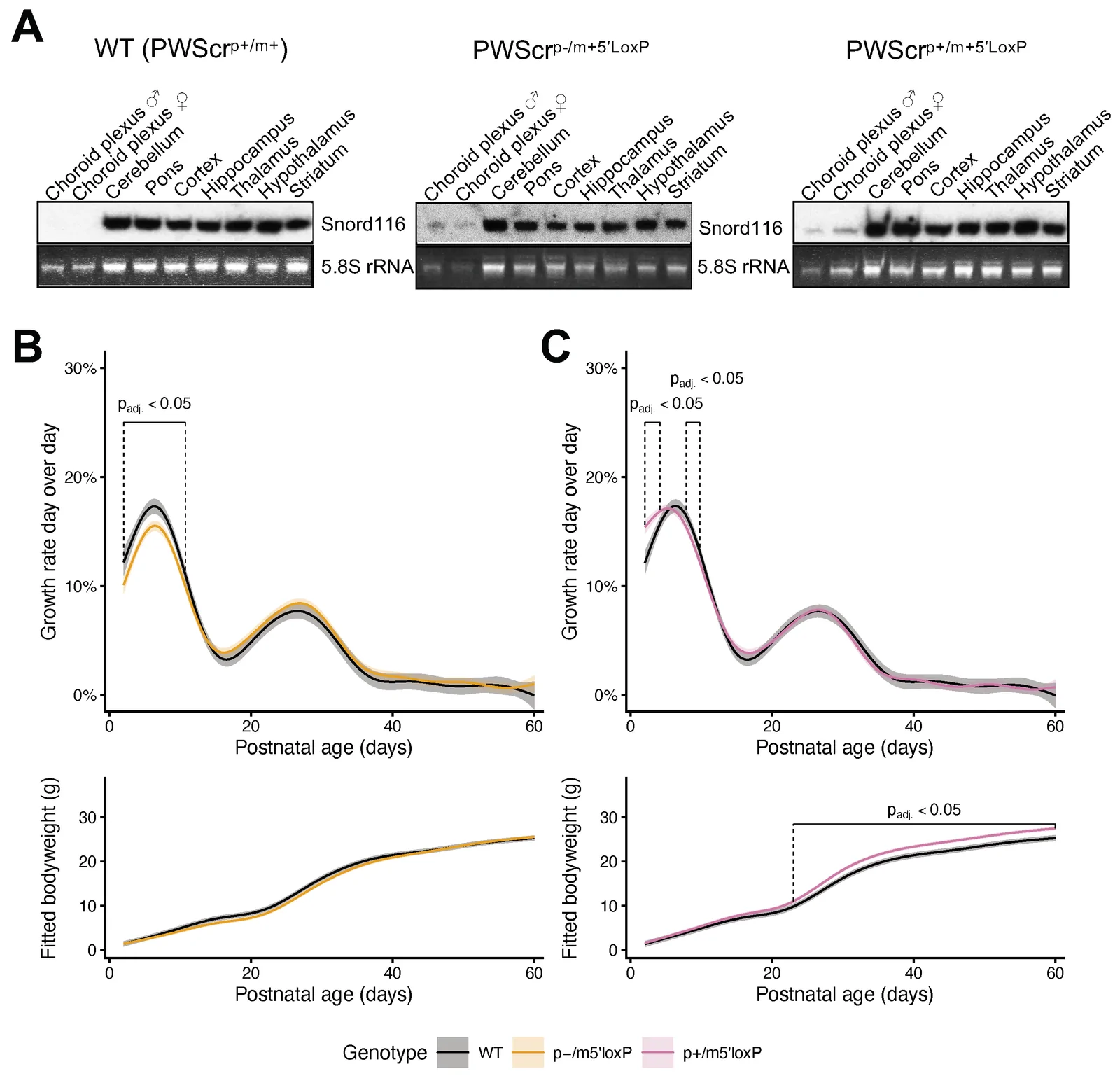
Snord116 expression in brain compartments and growth dynamics of male mice with maternal activation of the Snord116 cluster. (**A**) Northern blot analysis of Snord116 expression in brain compartments from wild-type (WT), PWScr^p−/m+LoxP^, and PWScr^p+/m+LoxP^ mice. The choroid plexus is a non-neuronal tissue; therefore, Snord116 expression in this compartment was detected only in mice harboring the maternally activated Snord116 cluster (PWScr^p−/m+LoxP^ and PWScr^p+/m+LoxP^). No sex-dependent differences in maternally activated Snord116 expression were observed. Ethidium bromide staining of 5.8S rRNA is shown as a loading control. (**B**,**C**) Growth dynamics of PWScr^p−/m+LoxP^ (**B**) and PWScr^p+/m+LoxP^ (**C**) male mice compared with WT control mice on the C57BL/6J genetic background. Upper panels show the relative day-to-day change in body weight (growth rate), and lower panels show longitudinal body-weight trajectories. Shaded areas represent the corresponding 95% confidence intervals for the fitted growth rate and bodyweight trajectories, respectively. Intervals in which differences between genotypes were statistically significant (*p*_adj_ < 0.05) are indicated by dashed lines.

In our previous study, we demonstrated that activation of the normally silent maternal PWS-critical region using an RNA polymerase II promoter containing a 5′LoxP cassette inserted upstream of the Snord116 cluster could compensate for the growth deficiency caused by the loss of the paternal PWS-critical region starting from postnatal day 21 in PWScr^p−/m+LoxP^ male mice [38]. Importantly, expression of the Snord116 cluster from the activated maternal allele was ubiquitous and approximately sevenfold lower than expression from the endogenous paternal allele (Figure 4A) [24,38]. Subsequently, we investigated the functional consequences of an increased dosage of Snord116-cluster transcription beyond physiological levels by analyzing mice carrying the 5′LoxP cassette on the maternal chromosome while also retaining an intact paternal allele (PWScr^p+/m+LoxP^); thus, simultaneously driving expression from both endogenous paternal and activated maternal allele (Figure 4A). Analysis of the postnatal growth rate revealed the distinct effects of maternal activation in the two genotypes. In the PWScr^p−/m+LoxP^ males, the daily growth rate was significantly lower during the early postnatal period (P2–P10) compared to the WT animals, with values approximately intermediate between those of the WT and Snord116KO mice (Figure 4B, upper left). However, compared with the Snord116KO animals, the PWScr^p−/m+LoxP^ mice rapidly recovered their growth rate and reached values comparable to the WT mice at around P10. From ~ P17, a small increase in growth rate compared to the WT mice was also observed, albeit it was not statistically significant. Consequently, the subsequent body-weight trajectories converged, and the PWScr^p−/m+LoxP^ animals approached the WT growth curve after P21 (Figure 4B, lower left). These observations are consistent with our previous finding that the relatively low expression level of the Snord116 cluster from the activated maternal allele is sufficient to substantially ameliorate the growth phenotype caused by the loss of paternal PWS-critical region expression [38]. However, it is important to note that mild but statistically significant differences in growth rate persisted during the first postnatal days.

In contrast, the PWScr^p+/m+LoxP^ males showed a distinct growth-rate profile. During the first four postnatal days, their relative growth rate was significantly higher than that of the WT animals, followed by a period of normalization and a subsequent phase of mildly reduced growth, with the difference in growth rate largely equalizing by approximately P10 (Figure 4C, upper right). Notably, an increase in body-weight gain compared with the WT animals was observed from approximately P16 to P19 (Figure 4C, upper right). The early differences in growth rate were reflected in the subsequent body-weight curve, with the PWScr^p+/m+LoxP^ males showing a modest but permanent increase in body-weight gain compared with the WT animals, even during later postnatal development (Figure 4C, lower right). These observations suggest that relatively small alterations in the growth rate during the earliest postnatal period may contribute to the subsequent changes in body weight trajectories between the PWScr^p+/m+LoxP^ and WT males, reaching statistically significant difference from P22 onwards (Figure 4C, lower right). Thus, as expected, activation of the maternal allele in the PWScr^p+/m+LoxP^ mice increases the dosage of the Snord116-cluster ncRNAs above the endogenous level, resulting in a modest increase in postnatal growth in the males. Interestingly, the effect of maternal Snord116-cluster activation was less pronounced in the females. Comparison of the PWScr^p+/m+LoxP^ and WT females revealed no differences in growth rate during the first postnatal days. A mild but statistically significant increase in growth rate was observed between P16 and P18; however, this transient increase did not result in a detectable difference in subsequent body-weight trajectories, which remained comparable between the PWScr^p+/m+LoxP^ and WT females (Figure S3C). Thus, the effect of the increased Snord116-cluster RNA dosage on postnatal growth appears to be modest and putatively sex-dependent, with the most apparent consequences observed in the PWScr^p+/m+LoxP^ males.

## 3. Discussion

The high temporal resolution of daily relative body-weight changes provides a useful framework for relating the Snord116-associated growth phenotype to the clinically defined developmental phases of human Prader–Willi syndrome. These comparisons should be considered as phenotypic correspondences rather than direct age-to-age equivalents because developmental timing, growth rates, and disease manifestations differ substantially between mice and humans (Figure 2A). Nevertheless, the progression of growth changes observed in the Snord116KO mice recapitulates several features of the developmental progression of PWS. At birth, Snord116KO mice do not show a major body-weight difference from their WT littermates, which is consistent with previous observations that the growth phenotype emerges postnatally rather than during late embryonic development [30,31,38]. Similarly, pronounced reduced birth weight is not a defining feature of human PWS, although prenatal and perinatal abnormalities can occur [47]. The most prominent phenotype in the Snord116KO mice occurs during the early postnatal period. Between ~ P2 and P16, mice show a marked reduction in relative body-weight gain, followed by a subsequent convergence of growth rates with the WT animals between ~ P17 and P21. This early growth-deficient period provides a relevant phenotypic parallel to the growth-deficient component of human PWS Phase 1a, which is characterized by hypotonia, weak suck, poor feeding, and inadequate weight gain during infancy. The subsequent stabilization of growth in the Snord116KO mice may be conceptually related to the transition towards more stable growth during human Phase 1b (Figure 2A–C) [2,4]. However, the much shorter developmental timescale in mice precludes assigning individual postnatal days to specific human ages (Figure 2A). The importance of the early postnatal period is also consistent with evidence that it represents a particularly sensitive growth window in mice, during which body weight increases rapidly and disruption of neonatal nutrition (starvation) can result in persistent growth deficits despite subsequent opportunities for catch-up growth [48,49,50].

Following this early growth deficit, Snord116KO mice undergo a transient increase in relative growth rate beginning at ~ P21. The duration of this increase differed slightly between genetic backgrounds, extending to ~ P33 in the case of the FVB × C57BL/6J background and to ~ P26 in the case of the C57BL/6J background (Figure 2B,C, Figures S1 and S2). This accelerated growth is consistent with a compensatory response to the preceding growth deficit, although it is insufficient to restore the body-weight trajectory to that of the WT animals. The transition from relatively stable growth to accelerated weight gain around P21 can be conceptually compared to the transition from the early growth phenotype towards the excess weight-gain phase (Phase 2a) of human PWS. In humans, Phase 2a can begin without a clear increase in appetite or food intake, but is followed by Phase 2b, when increasing interest in food and food intake accompany continued weight gain [1,2,8]. Notably, body-weight trajectories alone cannot establish the mechanism responsible for the increased growth rate after P21. The accelerated weight gain may reflect changes in energy expenditure, nutrient utilization, endocrine regulation, body composition, and/or other growth-regulatory processes rather than increased food intake alone. Consistent with the possibility of early metabolic alterations, endocrine analyses of the Snord116/Snord115 double-KO model revealed markedly reduced circulating insulin and leptin concentrations at P11 compared with the WT animals [23]. These observations support the view that the early growth phenotype is associated with systemic metabolic alterations, although the specific mechanisms linking Snord116 deficiency to altered growth remains to be established [6].

The comparison with human PWS also highlights an important limitation of the Snord116KO model. Human PWS can progress from Phase 2 into Phase 3, which is characterized by persistent hyperphagia and impaired satiety during childhood and a sustained risk of excessive weight gain and obesity in the absence of treatment and dietary control [1,2,47,51]. In contrast, the accelerated relative weight gain in the Snord116KO mice is transient, with growth rates returning to levels no longer significantly different from the WT mice after ~ P30. Thus, the model reproduces the early growth impairment and subsequent transition toward accelerated weight gain but does not reproduce the phenotype associated with human Phase 3. This difference may reflect species-specific developmental biology, differences in the contribution of the Snord116 cluster to the broader PWS phenotype, or the additional effects of neighboring genes and regulatory elements lost in typical PWS but retained in the Snord116KO model. In addition, the clinical course of individuals with PWS is strongly influenced by interventions such as growth hormone treatment, controlled nutrition, and restricted access to food, further limiting direct comparison with untreated experimental mice.

Furthermore, the reciprocal phenotype observed following activation of Snord116 from both parental and maternal alleles provides strong support for this interpretation. In contrast to the Snord116KO mice, these mice exhibit increased relative body-weight gain during the early postnatal period. Although their subsequent growth rate becomes more similar to that of the WT animals, the early growth advantage is retained as increased body weight during the later adult-life period in the male mice (Figure 4C). The two genetic models, therefore, reveal an inverse relationship between Snord116 dosage at early stages and subsequent body weight. Loss of Snord116 is associated with early growth impairment and a reduced body weight in adulthood, whereas increased Snord116 dosage is associated with an early growth advantage and a higher body weight. Expression from the reactivated maternal allele alone in the PWScr^p−/m5′loxP^ did not fully restore the early postnatal growth phenotype, despite substantially improving the later growth phenotype. This raises the possibility that the level of Snord116-cluster transcription achieved from the activated maternal allele [38], although sufficient to substantially rescue the later growth phenotype, may not be sufficient to fully recapitulate the early postnatal growth pattern observed in the WT animals or to completely prevent the early growth retardation, which is characteristic of the Snord116KO mice. This dose-dependent response might be evidence of a critical threshold of Snord116 abundance necessary for physiological function and should be taken into account in future experiments, e.g., transgenic compensation models or potential gene therapy.

Collectively, our findings have important implications for understanding the molecular basis of PWS. The developmental progression of the growth phenotype suggests that the consequences of Snord116-cluster deficiency are unlikely to be constant throughout postnatal development. Instead, the molecular processes underlying the phenotype may be particularly important during the early postnatal period and may subsequently engage different pathways as the phenotype evolves. Although, we have not investigated the impact of Snord116 loss during late embryonal stages, it is plausible that elimination of Snord116 could have functional consequence in the later embryonal stages, and this remains to be addressed [6,9]. Consequently, molecular analyses performed at a single postnatal age may miss the relevant transient molecular changes. The identification of distinct developmental postnatal phases in the present analysis, therefore, provides a rationale for a temporally resolved investigation of the Snord116KO mouse model, with particular focus on the earliest postnatal period and the subsequent transition toward an increased growth-rate phase. Such analyses may help distinguish early molecular alterations associated with the onset of the growth phenotype from later changes that may reflect secondary or compensatory responses, and may provide further insight into the molecular mechanisms contributing to PWS pathogenesis.

## 4. Materials and Methods

### 4.1. Animals

All mouse procedures were performed in accordance with the guidelines for the welfare of experimental animals issued by the Federal Government of Germany and were approved by the State Agency for Nature, Environment and Consumer Protection of North Rhine-Westphalia (LANUV; Landesamt für Natur, Umwelt und Verbraucherschutz Nordrhein-Westfalen). The animal procedures and experimental cohorts used in this study have been described previously [30,38]. No additional animals were generated or used specifically for the present study.

### 4.2. RNA Isolation

Total RNA was purified from mouse brain-tissue samples with the RNA Clean & Concentrator kit system (Zymo Research, Irvine, CA, USA, #R1017) using the Trizol (TRI Reagent, Zymo Research, #R2050-1-200) extraction method, according to the manufacturers’ instructions. Briefly, 800 µL of Trizol reagent was added to a −80 °C frozen sample, which was then homogenized, vortexed and incubated for 5 min at room temperature. Subsequently, 160 µL (0.2 × Vol of Trizol) of chloroform was added, and the mixture was vortexed thoroughly and spun for 10 min at 12,000× *g*. Notably, for isolation of total RNA from choroid plexus, 100 µL of Trizol reagent was used and the following protocol was modified accordingly. The resulting aqueous upper phase was removed to a new tube, mixed with the same volume of absolute ethanol and transferred to a column (Zymo-Spin IIC Column, Zymo Research, #C1011). The RNA was washed according to the protocol and eluted with 50 µL of ddH_2_O (25 µL of ddH_2_O for choroid plexus). Concentration was measured immediately with a NanoDrop spectrophotometer ND-1000 (Thermo Scientific, Waltham, MA, USA).

### 4.3. Northern Blot Analysis

Northern blot analysis was performed as described previously [24,30,38]. Briefly, for the analyses of ncRNA expression during mouse embryonal and early postnatal development (Figure 1B), approximately 20 µg of total RNA was denatured, fractionated on 1.2% agarose formaldehyde gels, and transferred to GeneScreen nylon membranes (NEN DuPont, Wilmington, DE, USA). Northern blot analysis of ncRNAs was performed with specific ^32^P-labeled oligonucleotides: Snord107: GCTCAATTTTTGAAATCATTATATTCAGACAAGG; Snord64: CTTCAGAGTAATCATTTTGAGCATCATTTC; Snord116: TTCCGATGAGAGTGGCGGTACAGA; and 5.8S rRNA: TCCTGCAATTCACATTAATTCTCGCAGCTAGC. In case of Snurf/Snrpn, RNA hybridization was performed with ^32^P-labeled cDNA probes. First strand cDNA synthesis was performed using Transcriptor reverse transcriptase (Roche, Basel, Switzerland) and SnrpnR1: CGATGCAGGCTATTAACAAACAA oligonucleotide followed by PCR amplification using, in addition, SnrpnD1: GACGCTTGGTTCTGAGGAGTGATT oligonucleotide. The cDNA probe was cloned in the pCRII vector (Invitrogen, Carlsbad, CA, USA), and subsequently sequenced. cDNA labeling was performed using random prime DNA labeling kit (Roche), and [α-^32^P] dCTP (PerkinElmer, Shelton, CT, USA).

During analyses of Snord116 expression in different mouse brain compartments (Figure 4A), 500 ng of total RNA was separated on a denaturing polyacrylamide gel (8% PAGE, 7 M Urea) and transferred onto a nylon membrane (BrightStar Plus, Ambion, Kaufungen, Germany, #AM10100). In case of choroid plexus samples, 200 ng of total RNA was used. Snord116 oligonucleotide probe (see above) was labeled with γ-^32^P-ATP using T4 polynucleotide kinase (New England Biolabs, Ipswich, MA, USA, #M0201). Membranes were first pre-hybridized for 30 min in 0.5 M sodium phosphate (pH 6.5), 7% (*w*/*v*) sodium dodecyl sulfate (SDS) buffer at 56 °C followed by overnight hybridization with specific probes at the same temperature. Membranes were washed twice at 46 °C in 2 X SSC buffer (20 mM sodium phosphate, 0.3 M NaCl, 2 mM EDTA, pH 7.4) containing 0.5% SDS for 30 min (washing was repeated when the blot showed high counts). Blots were exposed to MS-film (Kodak, Rochester, NY, USA) at −80 °C, and exposure time for each blot is indicated in the Figure legends.

### 4.4. Statistical Analysis

All statistical analyses were conducted in the R computational environment version 4.5.3 (2026-03-11) [52].

### 4.5. Calculation of Day-over-Day Growth Rate

Body weights of mice were measured daily for up to 60 days, starting from postnatal day 1 and going up to day 60 (Table S1). The day-over-day growth rate was calculated for consecutive days by subtracting the bodyweight of the preceding day from the current day and dividing the difference by the bodyweight of the preceding day. Therefore, the growth rate represents the proportional change in body weight from one day to the next. Measurements without a corresponding observation on the immediately preceding day were excluded from the growth-rate analysis.

### 4.6. Generalized Additive Mixed-Model Analysis

Differences in day-over-day growth rate between selected genotypes were analyzed using a generalized additive mixed model (GAMM) as implemented in the R package mgcv version 1.9.4 [53]. Genotype was included as a parametric fixed effect, while age was modeled using genotype-specific smooth functions such as growth_rate ~ genotype + s(age, by = genotype, k = 12), with random = list(Mouse_ID = ~1) as a mouse-specific random intercept accounting for repeated measurements of each animal. The model was fitted using restricted maximum likelihood (REML), and the smooth functions were fitted using 12 basis dimensions, allowing for potentially nonlinear age-dependent growth trajectories and differences in these trajectories between genotypes. To test the putative effect of within litter competition, the analysis was performed with inclusion of litter as a random effect of the GAMM (random = list(litter = ~1, Mouse_ID = ~1)) and compared with the fit of the original parameters above, which did not include litter (random = list(Mouse_ID = ~1)). The differences in the log-likelihoods of the two fitted models (without litter as random effect and with inclusion of litter) was analyzed via ANOVA.

### 4.7. Significance Testing

To characterize the age-dependent difference in growth rate between genotypes, model-based predictions were generated on a dense grid of 300 equally spaced time points spanning postnatal days 2 to 60. At each age, the difference between the predicted growth rates of the respective genotypes was calculated using the model’s linear predictor matrix with the mouse-specific random effect excluded, thereby representing the estimated genotype- and age-dependent growth trajectories independent of individual mouse effects.

In addition, simultaneous uncertainty bands for each age-dependent genotype contrast were calculated using the MASS package version 7.3.66 [54]. A multivariate normal simulation of 10,000 coefficient vectors was generated from the estimated model coefficient vector and its covariance matrix. For each simulation, the maximum absolute standardized contrast across all time points was calculated. The 95th percentile of this maximum statistic was used as confidence interval for each genotype contrast. This approach accounts for the correlation among predictions across age and provides simultaneous rather than discrete pointwise uncertainty intervals. Two-sided *p* values across the 300 time points were calculated using the corresponding Wald statistic and adjusted for multiple testing using the Benjamini–Hochberg (BH) method [55]. The growth-rate difference at any time point was considered statistically significant when BH adjusted *p* value was <0.05. For the growth-rate difference, the corresponding standard error of the respective contrast was estimated by combining the standard errors of the genotype-specific predictions, using the estimated difference ± 1.96 × its standard error as the 95% confidence interval.

### 4.8. Body-Weight Trajectory Analyses

Body-weight trajectories were analyzed using a linear mixed-effects model fitted by restricted maximum likelihood (REML) with the lmer function from the R package lme4 version 2.0.6 [56]. To account for potentially nonlinear changes in body weight with age, natural cubic splines with eight degrees of freedom were used in the model: bodyweight ~ genotype * ns(Age, df = 8) + (1 | Mouse_ID). Genotype, age, and their interaction were included as fixed effects, while mouse identity was included as a random intercept to account for repeated measurements. Genotype differences in body weight at each age were assessed by calculating estimated marginal means for each genotype at every unique age observed in the dataset using the emmeans package version 2.0.4. The resulting *p*-values were adjusted for multiple comparisons across ages using Holm’s method. Adjusted *p*-values below 0.05 were considered statistically significant.

## Figures and Tables

**Table 1:** Numbers of animals included in the study by genetic background, genotype, and sex. The numbers of male and female mice included in the study are shown according to genetic background and genotype. Wild-type (WT) and Snord116 knockout (Snord116KO) mice were analyzed based on the indicated genetic backgrounds. PWScr^p−/m+5′loxP^ and PWScr^p+/m+5′loxP^ mice were analyzed based on the C57BL/6J background. The total number of animals was 351, comprising 200 males and 151 females.

Background	Genotype	Number of Males	Number of Females
FVB/N × C57BL/6J	WT	28	24
FVB/N × C57BL/6J	Snord116KO	35	22
C57BL/6J	WT	21	20
C57BL/6J	Snord116KO	21	19
C57BL/6J	PWScr^p−/m+5′loxP^	45	27
C57BL/6J	PWScr^p+/m+5′loxP^	50	39
Total		200	151

## Data Availability

All data supporting the findings of this study are available within the manuscript and its Supplementary Materials.

## Supplementary Materials

The following supporting information can be downloaded at https://www.mdpi.com/article/10.3390/ijms27209344/s1.
