# Supplementary material for: Modeling the Prader–Willi Syndrome-Associated Growth Phases in Snord116 KO Mice
Source: Int J Mol Sci. 2026 Oct 21;27(20):9344. doi: 10.3390/ijms27209344 (PMC13705449; doi:10.3390/ijms27209344)
Supplement: Supplementary file 1 [file ijms-27-09344-s001.zip › original images.pdf]

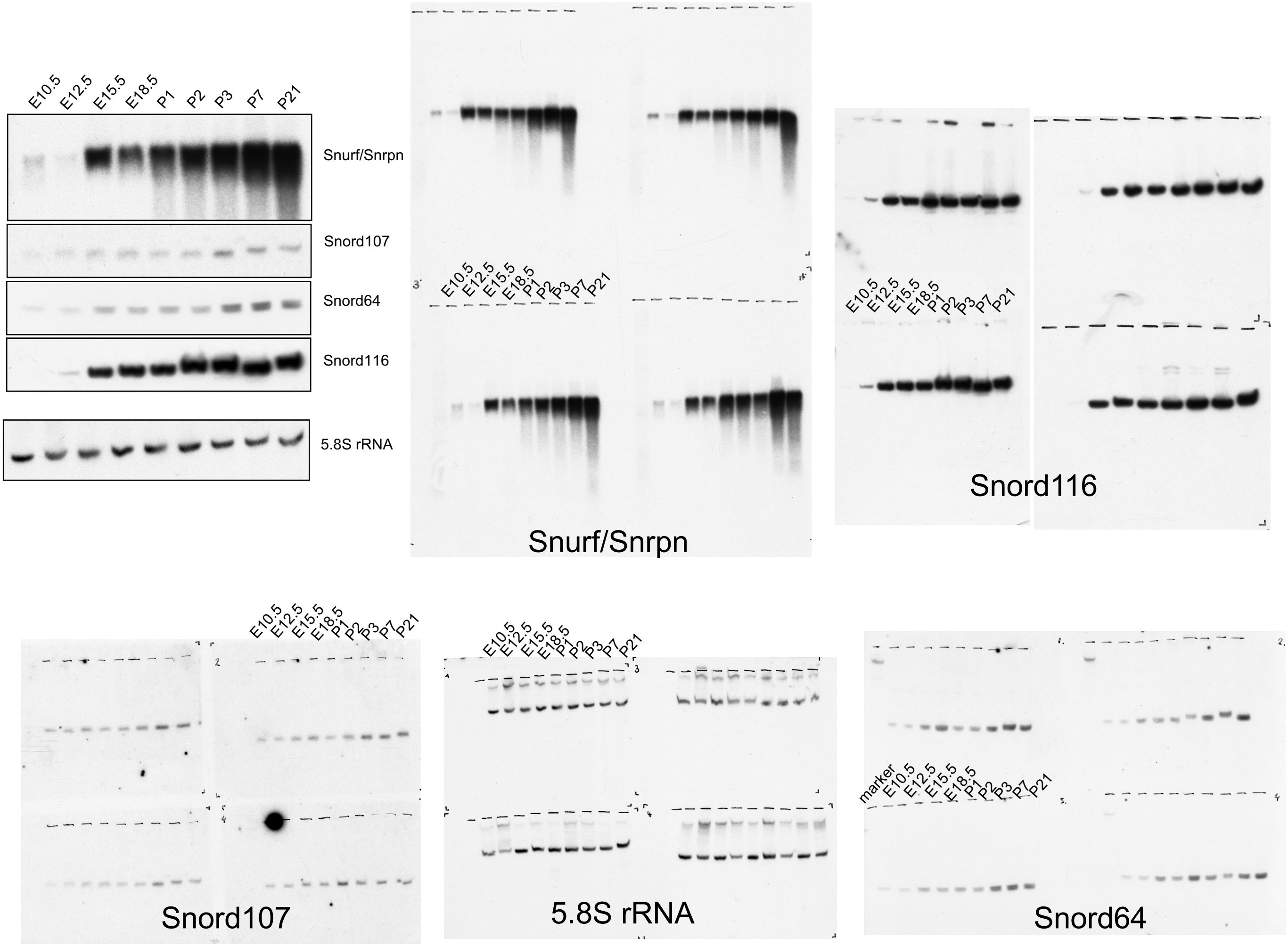

Figure 1B. Original Northern blot images. Figure 1B and multiple original Northern blot images are provided; the developmental stages corresponding to the images used to generate the figure are indicated.

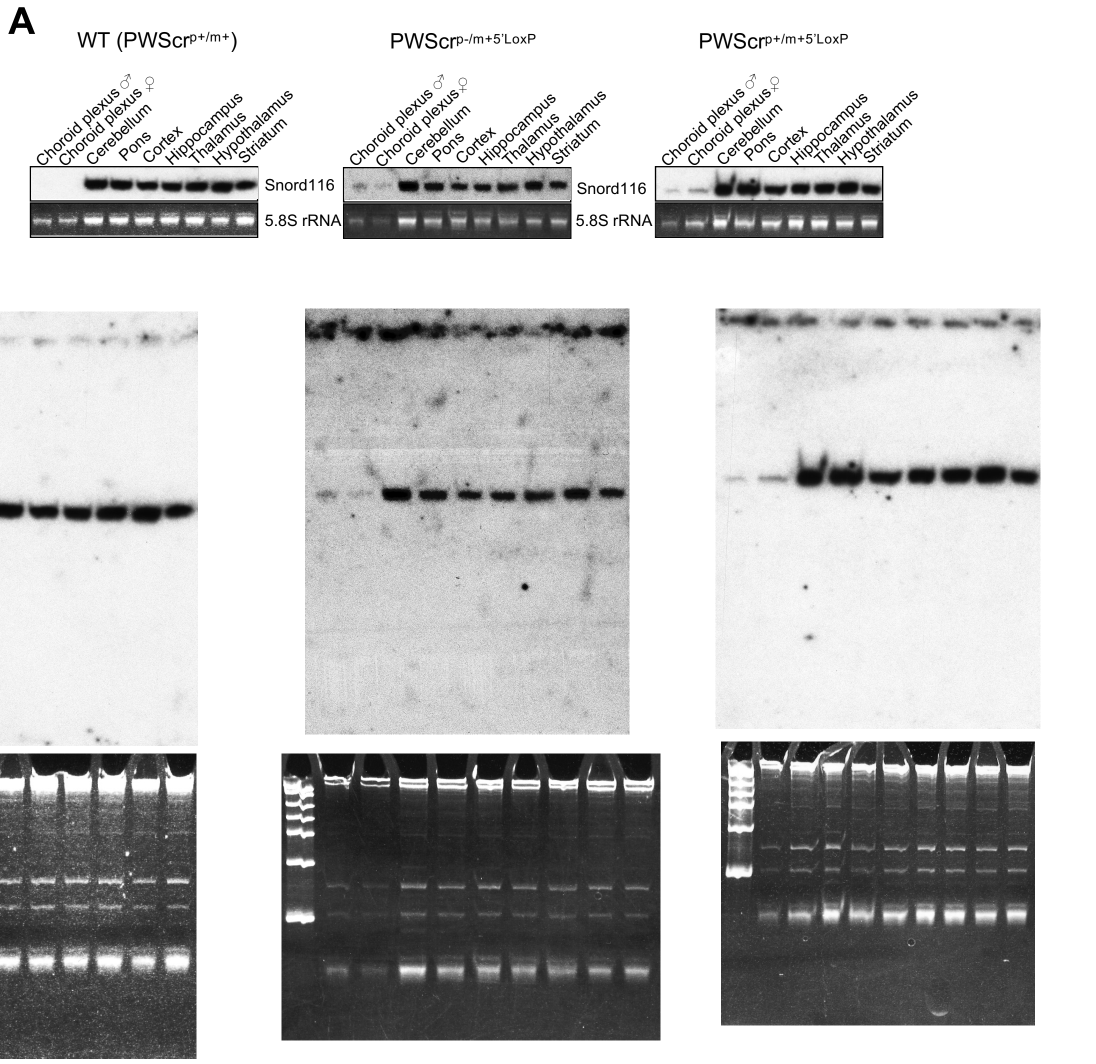

Figure 4A. Original Northern blot images and ethidium bromide-stained PAGE gels of total RNA from the corresponding samples. The cropped version of Figure 4A is shown at the top, with the corresponding original images shown below.
