# Supplementary material for: Modeling the Prader–Willi Syndrome-Associated Growth Phases in Snord116 KO Mice
Source: Int J Mol Sci. 2026 Oct 21;27(20):9344. doi: 10.3390/ijms27209344 (PMC13705449; doi:10.3390/ijms27209344)
Supplement: Supplementary file 1 [file ijms-27-09344-s001.zip › Supplementary Information.pdf]

Supplementary information

The Supplementary information file contains Figures S1-S3: Figure S1: Growth dynamics of Snord116 knockout mice of C57BL/6J genetic background; Figure S2: Statistically significant windows of growth-rate differences between Snord116-knockout and wild-type mice across sex and genetic backgrounds; and Figure S3: Growth dynamics of female mice with maternal activation of the Snord116 cluster.

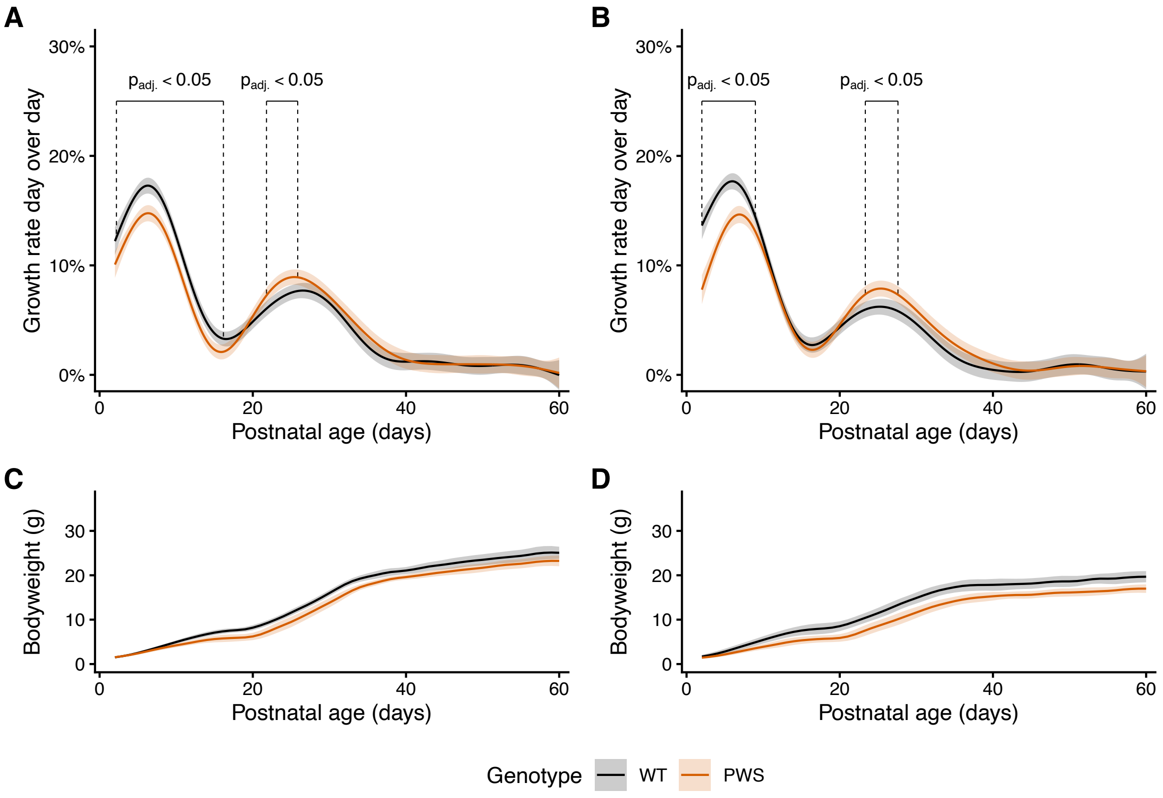

Supplementary Figure S1. Growth dynamics of Snord116 knockout mice of C57BL/6J genetic background.
(**A**, **B**) Growth dynamics in male (**B**) and female (**C**) Snord116KO and wild-type (WT) mice on the C57BL/6J genetic background. Upper panels show the relative day-over-day change in body weight (daily growth rate), whereas lower panels show longitudinal body-weight trajectories. Shaded areas represent the corresponding confidence intervals. Dashed boxes indicate developmental intervals in which differences in growth rates between genotypes were statistically significant after adjustment for multiple comparisons (Padj < 0.05).

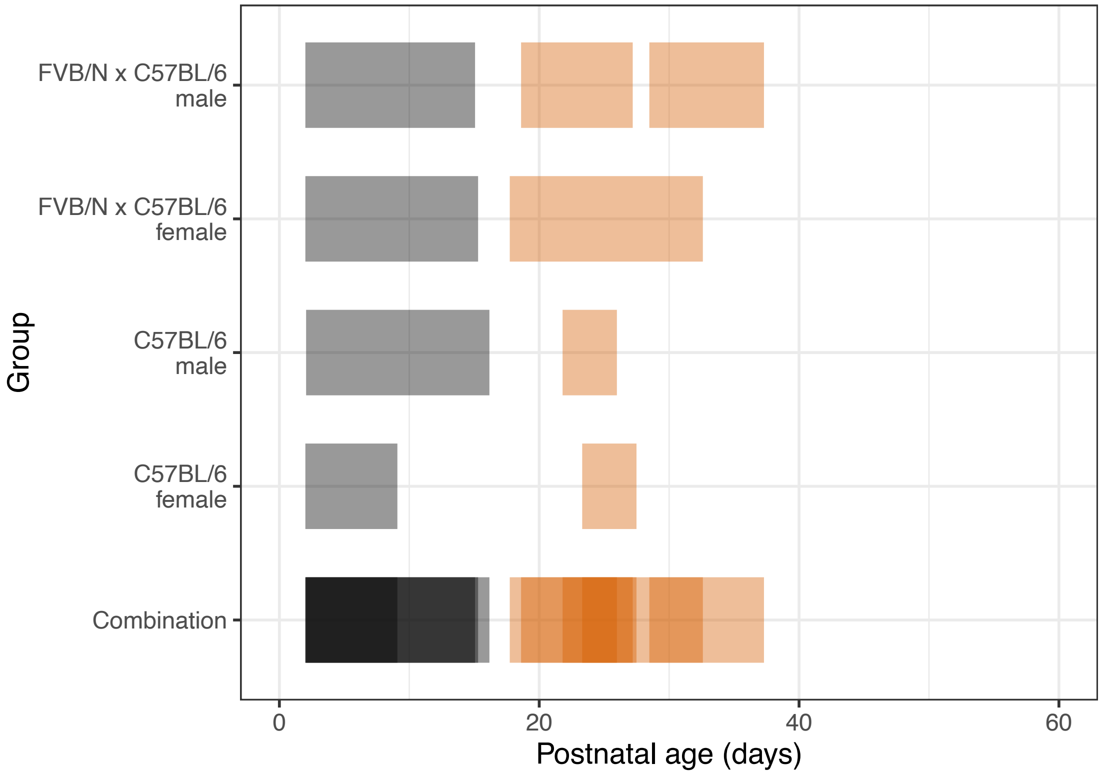

Supplementary Figure S2. Statistically significant windows of growth-rate differences between Snord116-knockout and wild-type mice across sex and genetic backgrounds. Statistically significant windows of growth-rate differences between Snord116-knockout (KO) and wild-type (WT) mice are shown separately for males and females across two genetic backgrounds (Figure 1B, C and Figure S1). Time windows during which KO and WT animals exhibited statistically significant differences in growth rate are indicated in grey and brown. The number of animals with the FVB×C57BL/6J genetic background was approximately 1.5-fold higher than that of the C57BL/6J cohort (Table 1), providing greater statistical power for the detection of differences in growth dynamics. Thus, the timing of significant growth-rate differences in the larger FVB×C57BL/6J cohort may provide more robust estimates of the developmental windows associated with altered growth in the Snord116-deficient model. Analyses were performed separately for each sex and genetic background to account for sex- and background-dependent differences in growth dynamics.

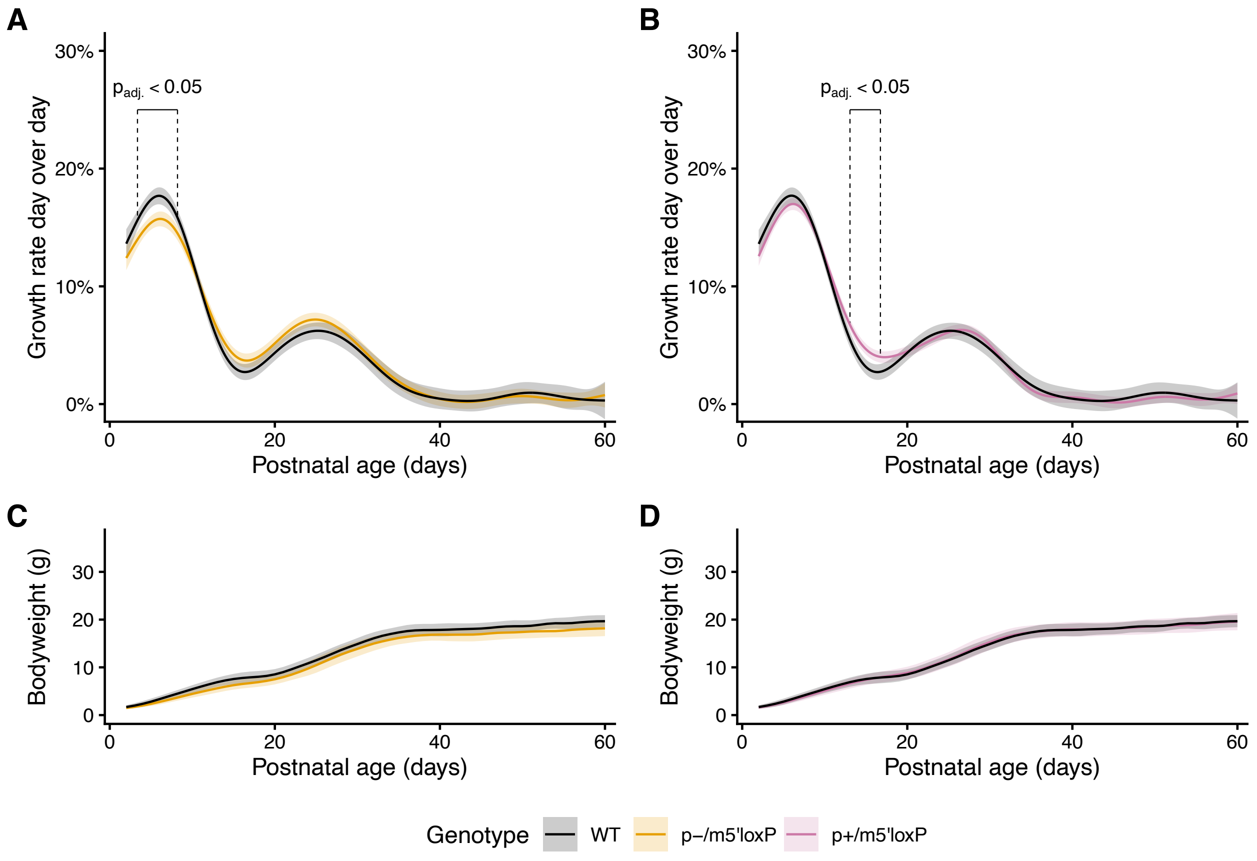

Supplementary Figure S3. Growth dynamics of female mice with maternal activation of the Snord116 cluster.

(**A** - **D**) Growth dynamics of $PWScr^{p-/m+LoxP}$ (**A, C**) and $PWScr^{p+/m+LoxP}$ (**B, D**) female mice compared with WT littermates on the C57BL/6J genetic background. **A**, **B** panels show the relative day-to-day change in body weight (daily growth rate), and **C**, **D** panels show longitudinal body-weight trajectories. Shaded areas indicate the corresponding confidence intervals. Dashed boxes denote developmental intervals during which differences in growth rates between genotypes reached statistical significance after correction for multiple comparisons (adjusted $P < 0.05$).
